# A Fungal Burn Infection

**Published:** 2014-02-21

**Authors:** Thomas R. Resch, Sean Main, Leigh Ann Price, Stephen M. Milner

**Affiliations:** Johns Hopkins Burn Center, The Johns Hopkins University School of Medicine, Baltimore, Md

**Keywords:** *Aspergillus*, burn, fungus, mold, pulse lavage

## DESCRIPTION

**Figure 1:**
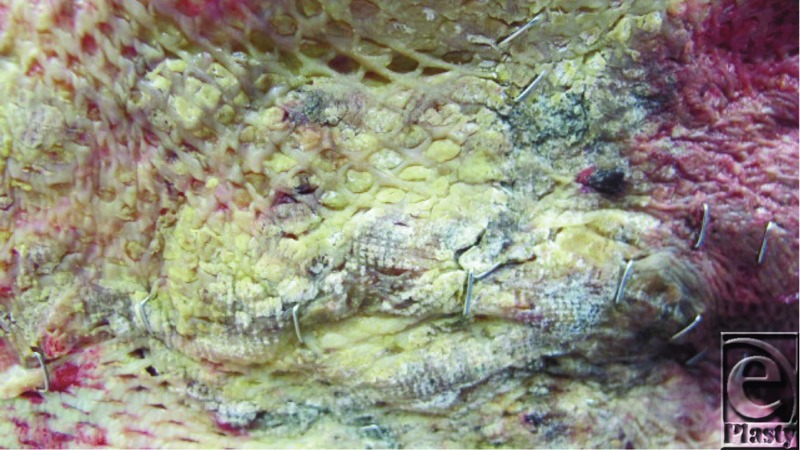
Burn wound on post-operative day three after resurfacing with allograft.

A 54-year-old man sustained third-degree burns to 50% total body surface area. He was transferred to our facility in septic shock on day five after injury. Broad-spectrum antibiotics, burn excision, and resurfacing with allograft were initiated. Two weeks later, a new wound infection was identified (Fig 1).

## QUESTIONS

1. **What is the differential diagnosis?**
2. **How is the diagnosis made?**
3. **What is the treatment?**
4. **What is the prognosis?**

## DISCUSSION

Confluent patches of a white-grey substance are evident both on and beneath the allograft (Fig 1). The broad-spectrum antibiotics suggest an opportunistic fungal infection. The differential diagnosis includes multiple pathogenic yeast and mold species. *Candida* spp are the most common burn wound colonizers and have historically been the most common invasive mycosis.^1^ Today, the most frequent invasive fungal pathogens include (in decreasing order): *Aspergillus, Fusarium, Candida, Mucor, Rhizopus, Microsporia*, and *Alternara* spp.^2^ Deadly non-albicans *Candida* spp such as *C. tropicalis and C. krusei* are also becoming more common.^3^

**Figure 2:**
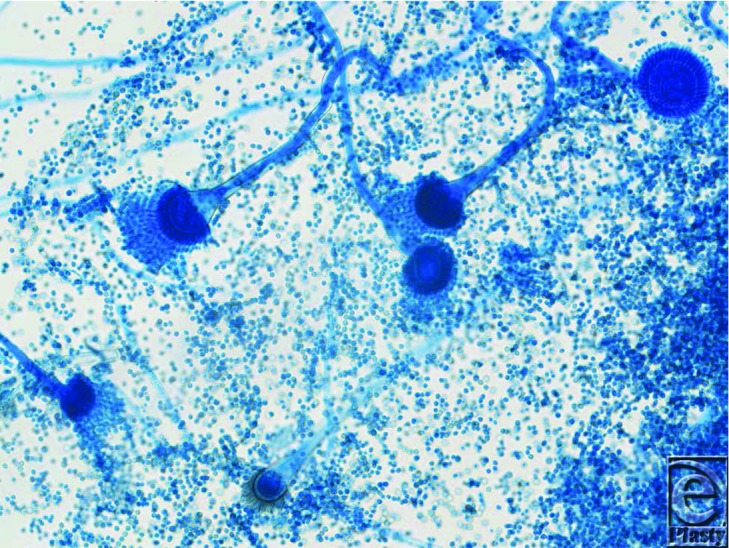
Light microscopy of *Aspergillus* spp with lactophenol cotton blue stain cultured from burn wound.

The diagnosis of an invasive fungal burn wound infection is most often made by the identification of microorganisms in viable tissue (below or adjacent to the burn wound) via biopsy.^1^^,^^2^ *Candida* infections may not always fit this criteria. Thus, concurrent wound and blood cultures yielding *Candida* spp should be substituted as evidence of an invasive infection.^1^ In this case, the fungal burden was so substantial as to be easily seen at the bedside (Fig 1). However, a more specific diagnosis (i.e. species) is difficult based upon clinical findings alone. In this case, a tissue sample was obtained from the area of greatest suspicion and expedited to the microbiology laboratory for histopathologic analysis. Invasive hyphae and spores characteristic of *Aspergillus* spp were identified and appropriate treatment was initiated. Wound cultures were also obtained and confirmed the presence of *Aspergillus* spp (Fig 2). It should be noted however, that fungal cultures (especially blood) are not always reliable and may require a long incubation time of one to two weeks before a definitive diagnosis can be made.^1^^,^^2^^,^^4^ Thus, if the diagnosis remains unclear, newer non-culture-based adjuncts such as a galactomannan EIA, (1→3)-β-D-glucans assay (Associates of Cape Cod Fungitell assay for invasive mycoses), or a PCR-based modality for *Aspergillus*-specific fungal genes should be considered.^5^

The invasive nature of molds such as *Aspergillus* spp mandate aggressive and prompt surgical excision until margins are histopathologically clear of fungal elements, in addition to the administration of systemic anti-fungal agents.^1^^,^^5^ Thus, treatment should not be delayed while awaiting culture results. Skin autografting should also be avoided until the infection has resolved.^3^ The systemic administration of targeted anti-fungal agents is important due to the angioinvasive nature of many molds such as *Aspergillus* spp. The first-line treatment is now Voriconazole, with Amphotericin B as second line due to its harsh adverse effects.^5^ Antifungal therapy alone is inadequate and must only be used in conjunction with surgical debridement.^1^ Currently, no topical antimicrobial agents have been found to be effective against invasive mold infections.^6^ Some silver based antimicrobial dressings have been shown to be successful at eradicating *Aspergillus* spp in vitro but have yet to be proven effective in vivo.^6^ In our case, the mold was successfully eradicated using a pulse lavage device with Amphotericin B solution to irrigate the wounds, in addition to surgical excision and systemic Voriconazole.

Infection with *Aspergillus* spp is associated with an especially high mortality. In one recent study, the mold accounted for 93% of all deaths attributed to fungus.^7^ When compared to other (non-mold) mycotic infections found in burn patients, *Aspergillus* spp are independently associated with a nearly 12-fold increase in the odds-ratio of death.^4^ This is most likely a reflection of the angioinvasive tendencies of the mold.

## References

[1] Becker WK, Cioffi WG, McManus AT (1991). Fungal burn wound infection: a 10-year experience. Arch Surg.

[2] Sarabahi S, Tiwari VK, Arora S, Capoor MR, Pandey A (2012). Changing pattern of fungal infection in burn patients. Burns.

[3] Ballard J, Edelman L, Saffle J (2008). Positive fungal cultures in burn patients: a multicentre review. J Burn Care Res.

[4] Spebar MJ, Lindberg RB (1979). Fungal infection of the burn wound. Am J Surg.

[5] Walsh TJ, Anaissie Ej, Denning DW (2008). Treatment of Aspergillosis: clinical practice guidelines of the Infectious Diseases Society of America. Clin Infect Dis.

[6] Wright JB, Lam K, Hansen D, Burrell RE (1999). Efficacy of topical silver against fungal burn wound pathogens. Am J Infect Control.

[7] Murray CK, Loo FL, Hospenthal DR (2008). Incidence of systemic fungal infection and related mortality following severe burns. Burns.

